# Pulsed Radiofrequency for Auriculotemporal Neuralgia: A Case Report

**DOI:** 10.3390/neurolint16020025

**Published:** 2024-03-12

**Authors:** Yan Tereshko, Enrico Belgrado, Christian Lettieri, Simone Dal Bello, Giovanni Merlino, Gian Luigi Gigli, Mariarosaria Valente

**Affiliations:** 1 Clinical Neurology Unit, Department of Head-Neck and Neurosciences, Udine University Hospital, Piazzale Santa Maria della Misericordia 15, 33100 Udine, Italy; 2 Department of Medicine (DMED), University of Udine, 33100, Udine, Italy

**Keywords:** pulsed radiofrequency, pain, auriculotemporal neuralgia

## Abstract

Auriculotemporal neuralgia is a rare facial pain disorder with no therapeutic evidence for refractory cases. We described a male patient with right auriculotemporal neuralgia, refractory to anesthetic nerve blocks and botulinum toxin type A injections, who was successfully treated with pulsed radiofrequency without adverse events. Pulsed radiofrequency may be an effective and safe treatment for refractory auriculotemporal neuralgia.

## 1. Introduction

The auriculotemporal nerve represents the terminal branch of the trigeminal nerve. Emerging from the foramen ovale outside the skull, the mandibular nerve, which is the third division of the trigeminal nerve, proceeds into the infratemporal fossa. Within this region, it bifurcates around the middle meningeal artery into two trunks: the anterior trunk and the posterior trunk. The latter further branches into the auriculotemporal nerve (ATn). The auriculotemporal nerve is responsible for providing cutaneous sensitivity to various areas, including the auriculotemporal region, external acoustic meatus, tragus, anterior portion of the ear, temporal scalp, posterior portion of the temple, tympanic membrane, temporomandibular joint capsule, and the parotid gland. Additionally, the auriculotemporal nerve carries parasympathetic fibers to the parotid gland [1].

Auriculotemporal neuralgia (ATN) is a rare disorder involving 0.23–0.4% of the patients in tertiary headache centers, characterized by unilateral side-locked pain localized in the territory of the auriculotemporal nerve [1,2,3,4]. The pain could be paroxysmal, moderate to severe in intensity, and variable in duration; however, continuous pain is often seen in this scenario. The pain can be spontaneous or can be triggered by pressure over the preauricular region, chewing, menses, gustatory stimuli, or facial tactile stimuli. The pain is usually throbbing (probably due to the temporal artery’s proximity) but could also be stabbing, lancinating, or shock-like and can radiate to the occipital, retro-orbital, or temporal region [2]. Some authors have proposed different mechanisms that can cause this disorder: compression at the level of the fascia bands in the preauricular region, the wrapping around the superficial temporal artery, the entrapment at the level of the pterygoideus externus muscle or between the pterygoideus internus and externus muscles, and mandibular overclosure [5,6,7,8,9]. This disorder may be misdiagnosed since the temporal and pulsatile localization may resemble the clinical features of migraine; in the case of auriculotemporal neuralgia, the localization is side-locked, photo-phono-osmophobia is typically absent, and the pain is triggered or exacerbated only by applying pressure over the preauricular region involved [1,2]. The auriculotemporal nerve is a branch of the trigeminal nerve; however, the pain quality and the localization of the pain are completely different from trigeminal neuralgia, and the efficacy of carbamazepine is not established [1]. Since this is a very rare disorder with clinical characteristics that overlap with other more frequent facial pain conditions, diagnosis and therapy could be challenging.

Anesthetic auriculotemporal nerve blockade is considered therapeutic and diagnostic; sometimes, the response is transient, and multiple nerve blocks are required. In a recent case series and case report, botulinum toxin type A (BoNT/A) was a valid alternative therapy in refractory cases [2,10]. The treatment of refractory cases is challenging, and the literature needs to be more conclusive. Pulsed radiofrequency (PRF) is a common treatment modality for chronic pain [11]. Pulsed radiofrequency (PRF) is a technique that applies radiofrequency electrical current to a target nerve; the electrical current is of short duration (20 ms) and is followed by a resting phase (480 ms) that repeats for a specific time [12,13,14]. Pulsed radiofrequency (PRF) generates an electromagnetic field to modulate the transmission of nerve impulses; the changes induced by electric fields act selectively on small, non-myelinated nerve fibers, producing a motor-sparing effect. Research has highlighted that the analgesic effect of PRF is not linked to thermal effects or permanent neural damage, suggesting a potential neuromodulatory mechanism. This mechanism may alter synaptic transmission or the excitability of C fibers. PRF affects afferent pathways and may exhibit a local anti-inflammatory effect by involving the immune system in the nociceptive process. Histological evaluations indicate that PRF induces transient endoneural edema, persisting up to a week after treatment. Pain relief commonly observed after PRF treatment can last for several months. Currently, PRF is used in the treatment of conditions such as radicular pain, occipital and trigeminal neuralgia, as well as shoulder and knee pain. Still, it is also effective in migraine, tension-type headaches, and cluster headaches [11,15,16,17,18,19]. Radiofrequency energy used in this technique is not continuous but pulsed, allowing for sufficient cooling to maintain a target temperature below 42 °C and prevent nerve axonotmesis or neurotmesis [20,21]. In a 2009 study, however, microscopic alterations were found in mitochondria, microfilaments, microtubules and membranes of C-fibers, A-delta fibers, and A-beta fibers [22]. This technique was never described in auriculotemporal neuralgia; however, it has been used in other neuralgias with promising results [18,19].

## 2. Case Description

We present a case of right auriculotemporal neuralgia, refractory to multiple nerve blocks and BoNT/A injections, successfully treated with pulsed radiofrequency (PRF). The patient is a 49-year-old man who came in May 2022 to our tertiary headache center complaining of persistent throbbing pain in the right preauricular and temporal region with superimposed spontaneous painful stabbing paroxysmal pain.

Sometimes, the pain radiated to the occipital region. The background pain was mild (NRS 3/10), and the exacerbations were severe (NRS 8/10); the exacerbations lasted 20–40 min and occurred 3–4 times per day. The pain started in February 2021, a few weeks after a mild SARS-CoV-2 infection. Neurological examination highlighted tenderness in the right temporal preauricular region with allodynia, and the pressure over the preauricular region determined acute stabbing pain along the course of the auriculotemporal nerve. He denied nausea, vomiting, photophobia, phonophobia, and osmophobia. Anesthetic right auriculotemporal nerve blockade with 1 mL of bupivacaine abolished pain after 5 min, and the effect lasted for 20 days; the procedure was performed five other times with the same result. Brain Magnetic Resonance Imaging (MRI) reported mild vascular gliosis, and angio-CT was normal; echography of the right temporal region excluded temporal artery involvement, and the MRI of the temporomandibular articulation did not show any pathologic changes. The dental exam was normal.

Blood tests were unremarkable. Oxygen 100% 12 L/min for 15 min and verapamil 80 mg TID were ineffective. We excluded cervicogenic headache, atypical facial pain, hemicrania continua, myofascial pain, temporomandibular junction dysfunction, tooth pain, chronic migraine, temporal arteritis, and trigeminal neuralgia based on the clinical features, neurological examination, and diagnostic instrumental tools. The anesthetic block of the auriculotemporal nerve was effective in aborting pain. However, the effect was not long-lasting. A diagnosis of right auriculotemporal neuralgia was performed. Since the disturb was refractory to anesthetic blocks, botulinum toxin type A therapy was discussed with the patient, and he accepted. We performed onabotulinumtoxinA subcutaneous injections (85 U) in the right preauricular and temporal region regions along the course of the auriculotemporal nerve; the therapy improved pain from a baseline of NRS 8/10 to 4/10 at 1-month evaluation and to 5/10 at 3-month evaluation; the frequency of the exacerbations remained the same and the background pain remitted. The benefit lasted about three months, and then the background and exacerbating pain returned to the baseline; the procedure was repeated twice with the same result. The outcome of onabotulinumtoxinA was satisfactory; however, it could not remit pain completely, and the patient’s quality of life was still impaired. We decided to perform pulsed radiofrequency with the patient’s consent; the patient was informed of the off-label nature of the therapy and the lack of any evidence in the literature regarding its efficacy in this clinical condition; he gave written formal consent for the treatment with pulsed radiofrequency and for his images and clinical information to be published.

## 3. Methods

**Figure 1:**
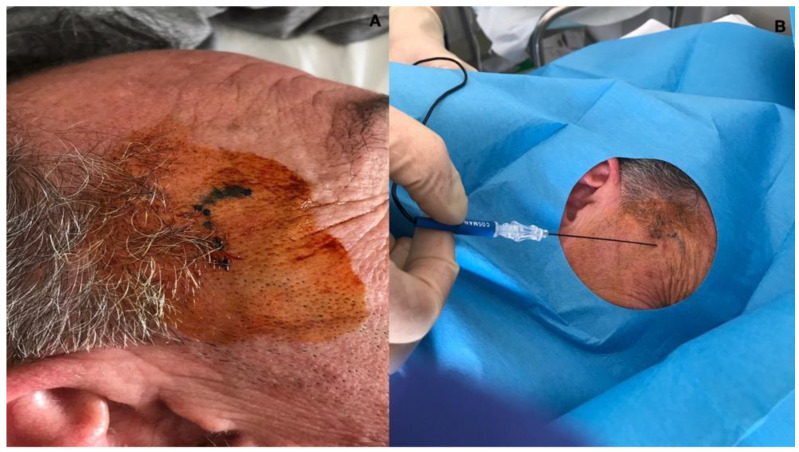
(**A**) shows the temporal area of the patient where the right superficial temporal artery was located with a landmark-guided technique. The auriculotemporal nerve was localized, drawing a line between the lateral cantus and the tragus; from the mid-point of this line, we proceeded upward, localizing the superficial temporal artery. The auriculotemporal nerve was about 0.5 cm anterior to the artery. (**B**) shows the RF needle inserted subcutaneously in the nearby of the right auriculotemporal nerve, anterior to the superficial temporal artery, with a cephalad direction and parallel to the auriculotemporal nerve. The 50 Hz sensory stimulation at 0.3 V determined the occurrence of paresthesia along the course of the auriculotemporal nerve and confirmed the correct stimulation site.

The procedure was performed using the Cosman G4^TM^ RF generator device (Cosman Medical Inc., Marlborough, MA, USA) and kit EchoRF^TM^. The patient was positioned in a prone position with the left aspect of the head lying on the bed, and disinfection with betadine was performed. We located the superficial temporal artery via palpation and inserted the RF needle (Cosman Medical Inc., Marlborough, MA, USA) (SCK 100 mm, 22G, curved, active tip 5 mm) 1 cm forward with the tip upward; when the needle was in position, the stylet was removed, and the RF probe was inserted (Figure 1). The correct position of the RF probe was confirmed using 50 Hz sensory stimulation at 0.3 V, which determined paresthesia in the territory of the right auriculotemporal nerve (the RF probe is at the target when the tingling sensation is evoked with a stimulation below 0.5 V). Then, a pulsed current was applied twice for 180 s (pulsed current for 20 ms at 2 Hz, followed by 480 ms of resting) with an 85 V output, an electrical current of 290 mA, and a 300 Ohm impedance. The temperature did not exceed 42 °C, preventing nerve damage.

## 4. Results

The background and the exacerbating pain gradually improved during the four weeks after the procedure, and at 1-month follow-up, there was a complete paroxysmal and background pain remission (NRS 0). Allodynia was remitted shortly after a 3-month follow-up. The pain remission persisted during the 6-month and 12-month follow-up and is still ongoing. The patient denied any adverse events related to the procedure and was satisfied with the long-standing positive outcome.

## 5. Discussion

Auriculotemporal neuralgia is not specifically addressed in the third edition of the International Classification of Headache Disorders [23]. In the second edition (ICHD-II), the auriculotemporal neuralgia (ATN) was considered as a possible inclusion under epigraph 13.7, which encompasses “other terminal branch neuralgias” of the trigeminal nerve. Consequently, in the current context, ATN does not align accurately with any diagnostic categories outlined in the third edition of the International Classification of Headache Disorders (ICHD-III) [1,2].

The neuralgia of the auriculotemporal nerve may stem from nerve entrapment during its passage through the lateral pterygoid muscle or at the level of the roof of the infratemporal fossa, frequently due to a muscle spasm or other pathologic conditions. Other potential causes include the formation of synovial cysts, malformations, aneurysms of the middle meningeal artery, and fractures of the mandibular condyle. Perineural spread of tumors involving the auriculotemporal nerve has also been reported [1,2]. In this case report, there were no secondary causes of auriculotemporal nerve neuralgia; it is probable that the etiology could be traced to the entrapment of the nerve during its passage through the lateral pterygoid muscle or at the level of the roof of the infratemporal fossa, the compression at the level of the superficial temporal artery, or could be idiopathic since the MRI was negative. Since our patients reported the occurrence of this disorder weeks after a SARS-CoV-2 infection, it could have had a possible role in its pathology. There have been some cases of trigeminal neuralgia probably due to a SARS-CoV-2 infection; however, the link between COVID-19 and our case of auriculotemporal neuralgia is only determined by the time that occurred between the infection and the clinical presentation of this disorder. Therefore, a certain connection is not feasible [24].

Given the rarity of this condition, the availability of high-quality studies is limited. The literature contains only a few articles discussing therapeutic options for auriculotemporal neuralgia. The evidence supporting treatments is primarily based on case series documenting positive responses to applying local anesthetic blocks of the auriculotemporal nerve, with or without steroids. Other mentioned therapeutic options include the use of medications such as carbamazepine and gabapentin or the use of botulinum toxin with controversial results [2,10,25]. Other treatment options may include cryoneuroablation and peripheral nerve stimulation, the latter used on male patients with chronic migraine pain in the distribution auriculotemporal nerve region [26,27]; however, these therapies were described only in single case reports and not on a large population of patients. To date, the most effective and reliable therapy is the anesthetic nerve blockade, while the other therapeutic options are still inconclusive. The treatments involving local anesthetic blocks of the auriculotemporal nerve and botulinum toxin injections yielded results for our patient that were not entirely satisfactory. Consequently, we decided to apply pulsed radiofrequency as a last resort since this approach successfully treats neuropathic pain conditions, and the short-term and long-term adverse events are very low [11,15,16,17,18,19].

Pulsed radiofrequency was never described in auriculotemporal neuralgia. However, this procedure has already been utilized in similar conditions such as occipital and trigeminal neuralgia [11]. Therefore, its application in other types of neuralgia, such as auriculotemporal nerve neuralgia, should be encouraged. This mini-invasive technique uses a pulsed current and maintains a target temperature below 42 °C, preventing nerve axonotmesis or neurotmesis [20,21]. The nerve stimulation affects pain by inducing long-term depression (LDT), inhibiting the C-fibers, and reducing pain signaling from the periphery to the central nervous system [28,29]. Moreover, it has been demonstrated that PRF enhances the descending inhibitory pathway [30]. On a molecular level, PFR exerts action on pain, decreasing dorsal horn microglia release of inflammatory cytokines and therefore reduces the facilitation in nociceptive signaling and sensitization [31,32,33]; moreover, it exerts its analgesic effect by the increase in mRNA expression of endogenous opioid precursors and related opioid peptides in the central nervous system [34].

Our patient improved after one PRF procedure and is still pain-free after twelve months, without any adverse events or nerve damage. No other cases reported in the literature were treated with PRF, and we hope our report may prompt others to use this therapy in this rare neuralgia and even in refractory cases. Moreover, it is unknown if the therapy may determine a complete remission of pain or may provide only temporary long-standing pain relief. In occipital neuralgia and trigeminal neuralgia, the literature reports temporary benefit; in the case of trigeminal neuralgia, a recent study applied PRF, CRF, and CCPRF to the Gasserian ganglion in a cohort of TN patients; the results in the PRF group were less satisfactory when compared to the other two groups, with pain relief of 82%, 9.1%, and 0% at 6-month, 12-month, and 24-month follow-up [35]. In most studies, the benefit lasted at least 3 months in occipital neuralgia [36].

Additional studies will be required to confirm the effectiveness of pulsed radiofrequency in managing auriculotemporal neuralgia, even though the rarity of this condition complicates the implementation of in-depth research.

## Data Availability

The original contributions presented in the study are included in the article, further inquiries can be directed to the corresponding author.

## Abbreviations

ATN	auriculotemporal neuralgia
BoNT/A	botulinum toxin type A
CT	computerized tomography
AEs	adverse events
NRS	numeric rating scale
MRI	magnetic resonance imaging
